# *CYP19A1* variation in healthy children: associations with breast development and pubertal gynecomastia

**DOI:** 10.1530/EC-26-0182

**Published:** 2026-09-01

**Authors:** Veronica L R Groendahl, Stine A Holmboe, Alexander S Busch, Casper P Hagen, Jørgen H Petersen, Trine H Johannsen, Kristian Almstrup, Hanne Frederiksen, Anders Juul, Lise Aksglaede

**Affiliations:** ^1^ Department of Growth and Reproduction, Copenhagen University Hospital – Rigshospitalet, Copenhagen, Denmark; ^2^ International Center for Research and Research Training in Endocrine Disruption of Male Reproduction and Child Health (EDMaRC), Rigshospitalet, University of Copenhagen, Copenhagen, Denmark; ^3^ Department of General Pediatrics, University of Münster, Münster, Germany; ^4^ Section of Biostatistics, University of Copenhagen, Copenhagen, Denmark; ^5^ Department of Clinical Biochemistry, Copenhagen University Hospital – Rigshospitalet, Copenhagen, Denmark; ^6^ Department of Cellular and Molecular Medicine, University of Copenhagen, Copenhagen, Denmark; ^7^ Department of Clinical Medicine, University of Copenhagen, Copenhagen, Denmark

**Keywords:** puberty, aromatase, thelarche, gynecomastia, genotypes

## Abstract

**Background:**

*CYP19A1* encodes aromatase, the enzyme converting androgens to estrogens, thereby influencing breast tissue development in healthy children.

**Objective:**

To examine associations between *CYP19A1* single nucleotide polymorphisms (SNPs) and circulating estradiol (E2) and testosterone (T), the E2/T ratio and age at thelarche in girls, and the presence of gynecomastia in boys.

**Methods:**

A total of 1,023 healthy participants (aged 5.9–20.0 years) from the Copenhagen Puberty Study were assessed for pubertal status, and serum sex steroid concentrations were measured by LC–MS/MS. Genotyping of *CYP19A1* SNPs (rs727479 A>C, rs2899472 A>C and rs10046 C>T) was performed.

**Results:**

Girls with the AA genotype in rs727479 and rs2899472 showed tendencies toward earlier pubertal onset. In a combined efficacy allele model, each additional allele was associated with 1.6 months earlier puberty (*P* = 0.06), and girls with 5–6 vs 0–2 efficacy alleles presented with significantly lower pubertal age (*P* = 0.02). The *CYP19A1* genotype was not associated with E2, T or E2/T levels. Boys with the CC genotype in rs727479 had the highest prevalence of pubertal gynecomastia (73%, *P* < 0.01). Boys with gynecomastia showed higher E2 and T (SDS) than unaffected boys (both *P* < 0.01), with no difference in E2/T. In age-matched analyses, E2 (SDS) remained elevated in boys with gynecomastia (0.81 vs 0.19, *P* = 0.04).

**Conclusion:**

*CYP19A1* variants may influence pubertal timing in girls. In boys, pubertal gynecomastia appeared to be linked with the *CYP19A1* genotype and was associated with elevated E2 concentrations.

**Significance statement:**

Aromatase, encoded by *CYP19A1*, catalyzes the conversion of androgens to estrogens. The contribution of common *CYP19A1* genetic variants to variation in pubertal development in healthy children is not fully understood. In this cohort from the Copenhagen Puberty Study, we examined associations between *CYP19A1* polymorphisms and circulating sex steroid concentrations, age at puberty and pubertal gynecomastia in boys. By integrating genetic, hormonal and clinical pubertal data, this study provides additional insights into potential links between aromatase-related genetic variation and normal pubertal development.

## Introduction

Age at pubertal onset varies substantially among healthy children. Genetic factors and racial differences explain a large part of this variation, but lifestyle and environmental factors also play an important role. The gold standard for assessing pubertal onset is breast development (Tanner stage B2) in girls and testicular enlargement above 3 mL in boys. However, in girls, breast development alone cannot determine whether Tanner stage B2 reflects central activation of the hypothalamic–pituitary–gonadal (HPG) axis or local estrogen production due to aromatase activity within the breast (1). Early pubertal development is associated with several adverse events later in life, such as an increased risk of cardiometabolic disease, breast cancer and mental health disorders (2, 3, 4). Therefore, the changes in timing of puberty have received considerable attention (5, 6).

Familial precocious puberty has been linked to rare monogenic variations in *DLK1, MKRN3, KISS1* and *KISS1R* (7, 8, 9, 10), and large population-based genome-wide association (GWA) studies have drawn attention to multiple genes related to altered pubertal timing with smaller effect sizes (11). More than 1,000 independent genetic loci have been associated with age at menarche (12); however, together they explain only about 10% of the variation in pubertal timing (13, 14, 15, 16, 17).

The enzyme aromatase is responsible for converting androgens to estrogens and is encoded by the *CYP19A1* gene, located on chromosome 15. Aromatase activity is present in various tissues in the body, such as the gonads, placenta, adipose tissue, bones, brain and eyes. Aromatase deficiency is a rare autosomal recessive condition associated with a reduced or absent conversion of androgens to estrogens, and a clinical presentation with unmeasurable or reduced concentrations of estrogens in combination with elevated concentrations of androgens and gonadotropins (18). In girls, aromatase deficiency may present at birth with virilized external genitalia and at the time of puberty with progressive virilization, delayed or absent puberty with lack of breast development and primary amenorrhea (19). In contrast, boys with aromatase deficiency may present in adolescence with absent pubertal growth spurt, delayed bone age due to lack of estradiol-induced epiphyseal fusion and, consequently, tall stature (20, 21, 22). Studies on specific *CYP19A1* single nucleotide polymorphisms (SNPs) in relation to pubertal onset are limited; nevertheless, one study indicated that TTTA repeat expansion in intron 4 of *CYP19A1* may be associated with precocious puberty in girls (23).

The *CYP19A1* SNPs rs727479 A>C and rs2899472 A>C have been linked to variation in circulating estradiol (E2) concentrations in adult populations, with the A alleles generally being associated with higher E2 and a higher risk of endometrial cancer (24, 25). Specifically, for rs727479, studies have reported that carriers of the C allele display lower circulating E2 compared with AA (26, 27, 28). Both rs727479 and rs2899472 are located in the intronic regions of *CYP19A1* and are hence not causing changes in the *CYP19A1* protein structure itself. In addition, variants in the 3′ UTR of *CYP19A1,* such as rs10046 C>T, have been associated with reduced concentrations of circulating E2 in postmenopausal women (29). Regarding the risk of estrogen-driven cancers, studies have shown conflicting results, with no certain increased risk associated with rs10046 variations (30).

In boys, pubertal gynecomastia is considered a physiological phenomenon commonly observed in mid-puberty at Tanner stages 3–4 and at testicular volumes of 5–10 mL. Gynecomastia occurs in approximately 50% of healthy boys, usually lasting for 6–24 months (31, 32). Pubertal gynecomastia may be caused by unbalanced conversion of T to E2, influencing glandular breast tissue growth (33). A recent study by Reinehr *et al.* reported an increased E2/T ratio in boys with gynecomastia compared with those without (34). Furthermore, studies suggest a role of leptin in the pathogenesis of pubertal gynecomastia. Elevated concentrations of leptin may directly stimulate mammary epithelial cells, enhance estrogen production by increasing aromatase activity and/or increase breast tissue sensitivity to estrogen (35).

In the present study, we evaluated sex- and age-related differences in circulating concentrations of E2 and T using sensitive LC–MS/MS methods in healthy girls and boys. Furthermore, we assessed three *CYP19A1* gene variants and their association with pubertal timing, the presence of gynecomastia, circulating concentrations of E2 and T and the E2/T ratio.

## Methods and materials

### Participants

Overall, 1,108 Danish children and adolescents examined in the Copenhagen Puberty Study (COPUS II) from 2006 to 2008 were eligible for this study. COPUS II was a combined cross-sectional and longitudinal study (36, 37). Only data from the cross-sectional part of COPUS II were included in this study. A total of 85 children were excluded due to lack of blood samples, leaving 1,023 children (58.4% girls) aged 5.9–20.0 years for analyses.

### Clinical examination

All participants underwent a clinical examination by trained physicians, including assessment of pubertal stages according to the Tanner classification (38, 39). In girls, breast development was assessed by palpation and breast stage B2 or above was considered a marker of pubertal onset. In boys, testicular volume was assessed by orchidometry and a testicular volume above 3 mL was considered a marker of pubertal onset (40). Gynecomastia was defined as subareolar proliferation of the glandular breast tissue. Pseudo-gynecomastia was defined as an increase in fatty tissue with no palpable glandular tissue (32).

Weight was measured to the nearest 0.1 kg using a calibrated digital electronic weight scale (Seca Delta, Germany, and Bisco Model PERS 200, Denmark), and height was measured to the nearest 0.1 cm using a wall-mounted stadiometer (Holtain Ltd, Crymych, United Kingdom). Skin fold measurements of subcutaneous fat layers (triceps, biceps, subscapular and flank) were performed with a fat-fold caliper (Harpenden, British Indicators Ltd, London, UK). Body fat percentage (BF%) was estimated using Slaughter’s equation for skinfold thickness (41).

### Hormone analyses

Blood samples were withdrawn from an antecubital vein and stored at −20°C until analysis.

Serum concentrations of total T and E2 were measured using highly sensitive, isotope-diluted online TurboFlow liquid chromatography–tandem mass spectrometry (LC–MS/MS) methods (42, 43). The limit of detection (LOD) was 0.031 nmol/L for T and 4 pmol/L for E2. T concentrations were below LOD in one girl (0.2%), whereas E2 concentrations were below LOD in 179 (36%) boys and in 71 (11%) girls. All analyses were performed at the Department of Growth and Reproduction, Rigshospitalet, Copenhagen, and accredited by the Danish Accreditation Fund in accordance with the DS/EN ISO15189 standard.

### Genotyping

Isolation of genomic DNA was performed using the Qiagen Nucleic Acid Isolation System (Qiagen, Germany) and quantified on a NanoDrop ND-1000 spectrophotometer (Saveen Werner, Limhamn, Sweden). All SNPs were analyzed at LGC Genomics (LGC Genomics, UK) using their KASP^TM^ SNP genotyping assays, which facilitate biallelic discrimination with a competitive PCR and incorporation of a fluorescent resonance energy transfer quencher cassette. KASP^TM^ genotyping assays were designed by LGC Genomics against the reference sequence containing the *CYP19A1* SNPs rs727479, rs2899472 and rs10046.

The Hardy–Weinberg equilibrium (HWE) was assessed for each SNP by comparing the observed genotype frequencies with the expected frequencies under HWE, calculated from the observed allele frequencies. Minor allele frequency (MAF) was calculated as the number of minor alleles divided by the total number of alleles (2n). HWE *P*-values were estimated using a chi-square goodness-of-fit test with 1 degree of freedom.

### Allele distribution

In girls, genotypes for rs727479 A>C were distributed as follows: AA = 68, AC = 290 and CC = 238 (MAF = 36%), consistent with HWE (*χ*^2^ = 3.71, *P* = 0.16).

For rs2899472 A>C, genotypes were distributed as follows: AA = 290, AC = 236 and CC = 69 (MAF = 31%), consistent with HWE (*χ*^2^ = 2.75, *P* = 0.25).

For rs10046 C>T, the distribution was as follows: CC = 154, TC = 307 and TT = 137 (MAF = 48%), consistent with HWE (*χ*^2^ = 1.19, *P* = 0.55).

In boys, genotypes for rs727479 A>C were distributed as follows: AA = 53, AC = 200 and CC = 171 (MAF = 36%), consistent with HWE (*χ*^2^ = 0.22, *P* = 0.90).

For rs2899472 A>C, genotypes were distributed as follows: AA = 254, AC = 148 and CC = 23 (MAF = 23%), consistent with HWE (*χ*^2^ = 0.06, *P* = 0.97).

For rs10046 C>T, genotypes were distributed as follows: CC = 98, TC = 219 and TT = 108 (MAF = 51%), consistent with HWE (*χ*^2^ = 0.41, *P* = 0.81).

### Statistical analyses

Differences in age and sex steroid hormone levels stratified according to each of the three *CYP19A1* SNPs were tested using the Kruskal–Wallis test. To estimate mean age (95% CI) at pubertal onset, probit regression models were applied, which correctly account for this type of current status data in which the exact age of B2 is unknown for everyone. Thus, prepubertal children (B1 in girls and testis volume <3 mL in boys) were treated as right-censored data, where only the lower bound for the true age of onset was recorded, whereas pubertal children (B2+ in girls and testis volume > 3 mL in boys) were treated as left-censored data, where only the upper bound for the true age of onset was recorded. To compare hormone levels between boys with and without gynecomastia, we performed age‐matched analyses using nearest-neighbor matching with a 1:1 ratio. Weighted linear models and nonparametric sensitivity tests were then applied to account for the matched design and evaluate group differences.

For genetic stratification, subjects were grouped based on ‘efficacy alleles’ – allelic variants previously linked to functional differences in enzyme activity or hormonal response, such as increased circulating E2 concentrations. We defined the A alleles of rs727479 and rs2899472, along with the T allele of rs10046, as efficacy alleles, based on previous evidence from postmenopausal populations linking these variants to altered circulating E2 levels. Individuals were categorized into three groups according to their total number of efficacy alleles (0–2, 3–4 and 5–6), enabling comparison of pubertal timing and hormone levels across these strata. This combined efficacy allele analysis was conducted as a pre-specified exploratory analysis, summarizing cumulative genetic variation within the *CYP19A1* locus under a hypothesis-driven additive model.

For statistical calculations and graphical illustration, hormone values below LOD were replaced with LOD/2. The threshold for statistical significance was set at *P* < 0.05. The data were analyzed using R, version 4.2.0, and SPSS, version 29.0.1.

To enable comparisons across sex and ages, concentrations of reproductive hormones were standardized to age-related standard deviation scores (SDSs) using the following equation: SDS = ((X/M)^^L^ −1)/(L × S), with X being the measurement, M corresponding to the median, L adjusting for skewness (*L* ≠ 0) and S approximating the coefficient of variation as published by Frederiksen *et al.* (42, 43).

All analyses of pubertal timing and hormone levels were adjusted for BF% and BMI.

## Results

### Impact of *CYP19A1* variants on circulating concentrations of E2 and T and the E2/T ratio and height

**Figure 1:**
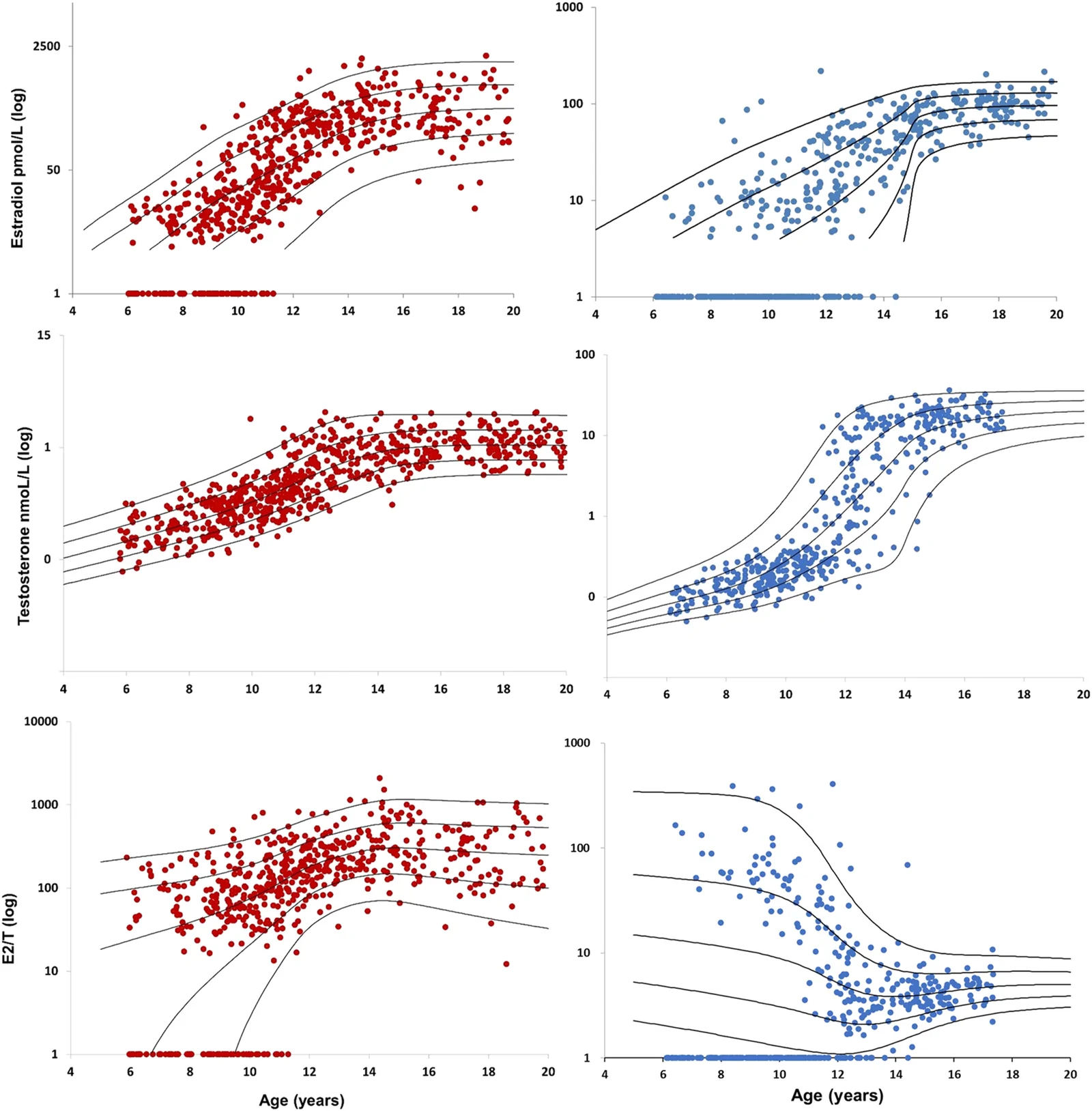
Concentrations of E2 and T and the E2/T ratio in girls and boys. Serum concentrations of estradiol (E2) and testosterone (T) and the E2/T ratio in healthy children aged 6–20 years. Concentrations are shown on a logarithmic scale and plotted separately for girls (in red) and boys (in blue) according to age. Each point represents a measurement, and the lines indicate mean and ±2 standard deviations. *Boys n = 424; girls n = 599.*

Serum concentrations of E2 and T and the E2/T ratio according to sex and age are illustrated in Fig. 1. No effects on circulating hormones and BF % according to *CYP19A1* genotypes were found, as shown in Table 1 (girls) and Table 2 (boys). Height SDSs did not differ significantly across *CYP19A1* genotypes in either girls or boys.

**Table 1:** Sex hormones, body fat percentage and height according to CYP19A1 genotypes in girls.

		**rs727479**		
	**AA**	**AC**	**CC**	***P-*value** *
***n***	***68***	***290***	***238***	
Age (years)	11.9 (10.3, 14.9)	12.2 (10.3, 15.7)	12.0 (10.1, 15.3)	0.69
E2 (pmol/L)	51.2 (11.8, 209.0)	103.3 (16.0, 244.6)	64.8 (14.5, 229.9)	0.26
E2 (SDS)	−0.17 (−0.66, 0.62)	0.11 (−0.54, 0.94)	−0.09 (−0.59, 0.62)	0.10
T (nmol/L)	0.5 (0.2, 0.9)	0.5 (0.3, 1.0)	0.4 (0.2, 1.0)	0.30
T (SDS)	−0.03 (−0.75, 0.63)	0.17 (−0.52, 0.84)	0.00 (−0.80, 0.71)	0.22
E2/T	137.3 (67.3, 232.6)	161.0 (75.0, 304.3)	144.0 (57.0, 290.5)	0.32
E2/T (SDS)	0.39 (−0.65, 1.36)	0.42 (−0.53, 1.26)	0.41 (−0.44, 1.20)	0.98
Body fat (%)	18.8 (11.0, 29.3)	19.8 (11.4, 30.4)	18.9 (11.8, 30.1)	0.64
Height (SDS)	−0.01 (−0.21, 0.19)	−0.06 (−0.17, 0.05)	−0.05 (−0.19, 0.09)	0.94
		**rs2899472**		
	**AA**	**AC**	**CC**	
***n***	***290***	***236***	***69***	
Age (years)	12.2 (10.1, 14.9)	11.6 (9.8, 15.7)	13.1 (11.3, 16.8)	0.21
E2 (pmol/L)	92.2 (16.8, 254.8)	49.0 (12.1, 215.3)	133.2 (22.2, 243.0)	0.34
E2 (SDS)	0.09 (−0.54, 0.89)	−0.06 (−0.65, 0.73)	−0.13 (−0.60, 0.65)	0.91
T (nmol/L)	0.5 (0.2, 1.0)	0.4 (0.2, 0.9)	0.7 (0.3, 1.1)	0.22
T (SDS)	0.03 (−0.80, 0.75)	0.17 (−0.53, 0.77)	−0.09 (−0.75, 0.75)	0.87
E2/T	164.7 (76.6, 310.8)	125.5 (50.0, 260.8)	166.6 (91.2, 263.5)	0.37
E2/T (SDS)	0.31 (−0.69, 1.21)	0.55 (−0.27, 1.39)	0.57 (−0.23, 1.04)	0.99
Body fat (%)	19.2 (11.8, 29.6)	19.8 (11.4, 30.4)	18.2 (10.8, 30.8)	0.85
Height (SDS)	−0.05 (−0.16, 0.06)	−0.08 (−0.22, 0.05)	0.01 (−0.21, 0.25)	0.76
		**rs10046**		
	**CC**	**TC**	**TT**	
***n***	***154***	***307***	***137***	
Age (years)	12.0 (10.0, 15.4)	12.0 (10.2, 15.7)	12.3 (10.5, 14.7)	0.98
E2 (pmol/L)	48.3 (15.0, 230.0)	89.8 (14.4, 231.2)	107.0 (18.5, 228.9)	0.53
E2 (SDS)	−0.21 (−0.6, 0.68)	0.12 (−0.55, 0.84)	−0.07 (−0.57, 0.71)	0.32
T (nmol/L)	0.5 (0.1, 1.6)	0.5 (0.1, 1.6)	0.5 (0.1, 1.8)	0.51
T (SDS)	−0.13 (−1.7, 1.5)	0.14 (−1.47, 1.62)	−0.01 (−1.78, 1.93)	0.18
E2/T	144.01 (56.45, 260.58)	160.91 (59.1, 305.08)	140.16 (82.39, 293.01)	0.57
E2/T (SDS)	0.46 (−0.35, 1.02)	0.41 (−0.50, 1.38)	0.35 (−0.65, 1.21)	0.83
Body fat (%)	19.2 (11.3, 30.0)	19.3 (11.9, 30.7)	19.1 (11.5, 29.4)	0.93
Height (SDS)	−0.06 (−0.23, 0.12)	−0.07 (−0.18, 0.04)	−0.01 (−0.17, 0.14)	0.85

*P-value < 0.05 was considered statistically significant.

**Table 2:** Sex hormones, body fat percentage and height according to CYP19A1 genotypes in boys.

		**rs727479**		
	**AA**	**AC**	**CC**	***P-*value** *
***n***	***53***	***200***	***171***	
Age (years)	12.3 (9.4, 15.3)	13.2 (10.7, 16.0)	12.4 (10.1, 14.8)	0.10
E2 (pmol/L)	8.0 (2.0, 66.6)	32.0 (2.0, 78.3)	20.4 (2.0, 70.7)	0.20
E2 (SDS)	0.04 (−0.41, 0.59)	0.14 (−0.44, 0.71)	0.17 (−0.30, 0.74)	0.40
T (nmol/L)	0.8 (0.2, 15.3)	7.0 (0.3, 17.1)	2.4 (0.2, 16.7)	0.20
T (SDS)	−0.09 (−0.59, 0.47)	0.09 (−0.68, 0.83)	0.24 (−0.60, 1.03)	0.21
E2/T	7.1 (4.5, 13.2)	5.5 (3.8, 11.0)	6.4 (3.7, 14.1)	0.30
E2/T (SDS)	0.43 (−0.31, 0.92)	0.44 (−0.19, 1.03)	0.46 (−0.04, 0.92)	0.96
Body fat (%)	15.3 (9.2, 29.1)	15.3 (8.8, 29.8)	16.0 (8.5, 32.4)	0.58
Height (SDS)	0.16 (−0.12, 0.44)	0.02 (−0.13, 0.17)	0.02 (−0.15, 0.19)	0.68
		**rs2899472**		
	**AA**	**AC**	**CC**	
***n***	***254***	***148***	***23***	
Age (years)	12.6 (10.7, 15.7)	12.7 (10.6, 15.5)	11.4 (9.4, 14.5)	0.21
E2 (pmol/L)	26.6 (2.0, 73.8)	26.6 (2.0, 75.8)	2.0 (2.0, 51.1)	0.34
E2 (SDS)	0.14 (−0.34, 0.72)	0.13 (−0.40, 0.74)	0.11 (−0.31, 0.60)	0.91
T (nmol/L)	5.3 (0.2, 17.1)	4.0 (0.3, 16.4)	0.3 (0.2, 12.2)	0.22
T (SDS)	0.06 (−0.63, 0.97)	0.09 (−0.63, 0.89)	0.00 (−0.69, 0.62)	0.87
E2/T	6.0 (4.1, 12.0)	5.5 (3.6, 13.4)	9.4 (4.4, 17.0)	0.37
E2/T (SDS)	0.43 (−0.19, 1.00)	0.45 (−0.04, 0.92)	0.53 (−0.05, 0.87)	0.98
Body fat (%)	15.0 (8.6, 32.3)	16.1 (8.9, 31.6)	15.4 (7.9, 23.5)	0.36
Height (SDS)	0.07 (−0.07, 0.21)	0.02 (−0.15, 0.19)	−0.20 (−0.62, 0.23)	0.68
		**rs10046**		
	**CC**	**TC**	**TT**	
***n***	***98***	***219***	***108***	
Age (years)	12.9 (10.7, 15.2)	12.4 (10.4, 15.7)	12.4 (10.3, 15.3)	0.57
E2 (pmol/L)	30.0 (2.0, 75.4)	27.0 (2.0, 77.6)	15.0 (2.0, 64.9)	0.42
E2 (SDS)	0.15 (−0.32, 0.70)	0.13 (−0.37, 0.80)	0.15 (−0.38, 0.64)	0.98
T (nmol/L)	5.6 (0.3, 17.2)	4.6 (0.3, 16.8)	2.3 (0.2, 15.3)	0.35
T (SDS)	0.24 (−0.46, 1.02)	0.09 (−0.71, 0.89)	−0.06 (−0.56, 0.77)	0.53
E2/T	5.3 (3.4, 12.4)	6.0 (4.1, 13.1)	6.4 (4.0, 12.6)	0.46
E2/T (SDS)	0.52 (0.06, 0.88)	0.46 (−0.11, 1.03)	0.36 (−0.51, 0.89)	0.29
Body fat (%)	15.4 (8.2, 30.9)	15.5 (8.8, 32.2)	15.3 (9.1, 31.2)	0.98
Height (SDS)	−0.01 (−0.22, 0.21)	−0.04 (−0.10, 0.20)	0.05 (−0.15, 0.26)	0.90

*P-value < 0.05 was considered statistically significant.

### *CYP19A1* genotypes and pubertal timing

#### Girls

**Figure 2:**
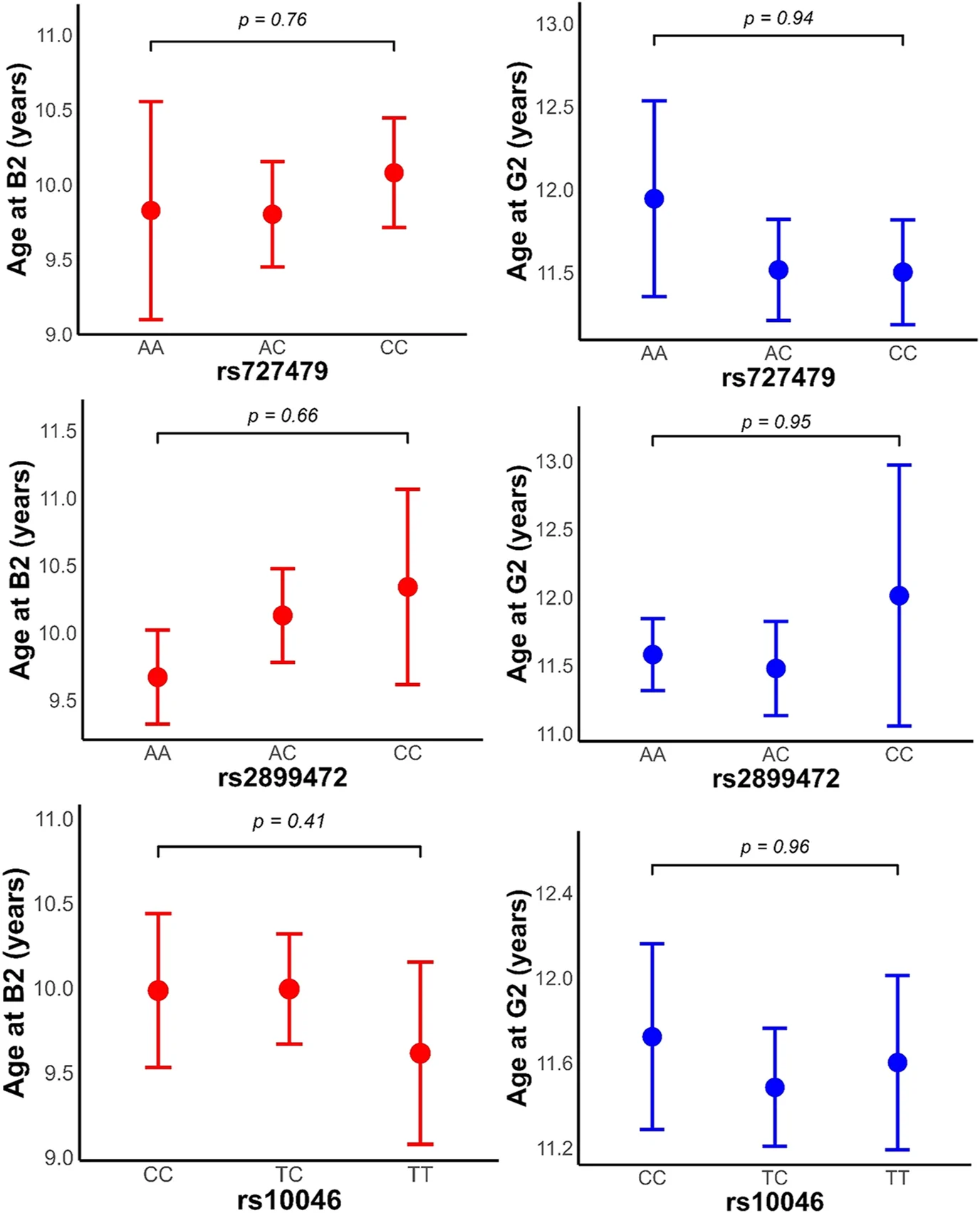
Pubertal onset according to *CYP19A1* genotypes in girls and boys. Model-derived estimates of mean age at thelarche in girls (red) and age at pubertal onset in boys (blue) according to *CYP19A1* genotypes (rs727479, rs2899472 and rs10046). Estimates were derived from probit regression models applied to current status data, with prepubertal participants treated as right-censored and pubertal participants as left-censored observations. The error bars indicate 95% confidence intervals. *P*-values reflect p-trend across genotype groups. Pubertal onset was defined as thelarche/breast stage ≥2 in girls (B2) and testicular volume >3 mL in boys (G2). *Boys n = 424; girls n = 599.*

Figure 2 shows the association between *CYP19A1* genotypes and estimates of mean age at pubertal onset.

For the rs727479 (A>C) SNP, the mean age at pubertal onset was 9.61 years for girls with the AA genotype, 9.75 years for those with the AC genotype and 9.98 years for those with the CC genotype (*P*-trend = 0.54).

For rs2899472 (A>C), the corresponding ages were 9.67 years (AA), 9.93 years (AC) and 10.20 years (CC) (*P*-trend = 0.36).

For rs10046 (C>T), pubertal onset occurred at 9.46 years (TT), 9.92 years (TC) and 9.91 years (CC) (*P*-trend = 0.28).

**Figure 3:**
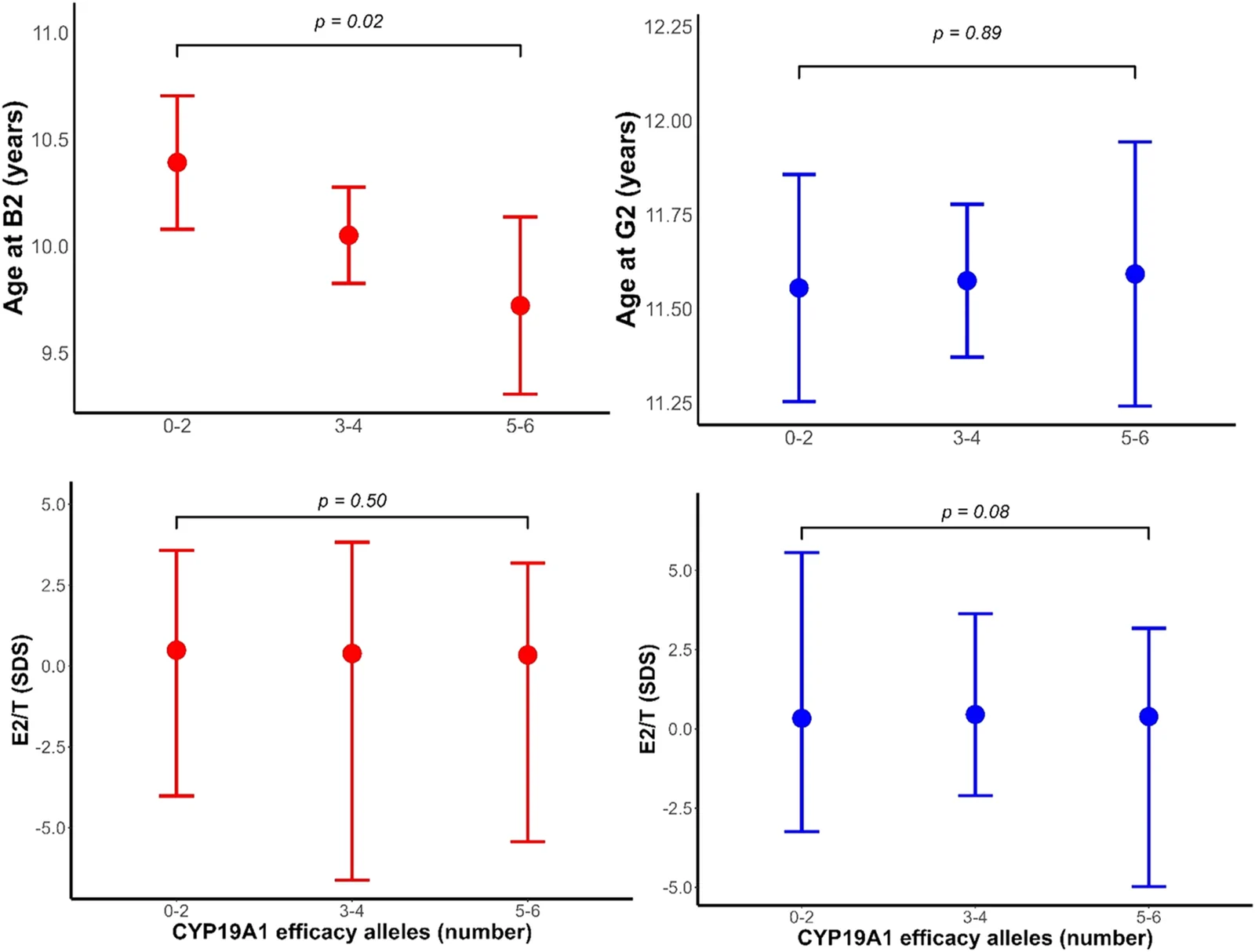
Pubertal timing and the E2/T ratio by the number of *CYP19A1* efficacy alleles in girls and boys. Model-derived estimates of mean age at thelarche in girls (red) and pubertal onset in boys (blue) according to the total number of *CYP19A1* efficacy alleles (0–2, 3–4 and 5–6) across rs727479, rs2899472 and rs10046 (upper panel). Estimates were derived from probit regression models. The E2/T ratio according to the efficacy allele group is shown in the lower panel, and differences were assessed using the Kruskal–Wallis test. The error bars indicate 95% confidence intervals. *Boys n = 424; girls n = 599.*

Figure 3 presents the combined efficacy allele model across the three SNPs. In this exploratory analysis, each additional efficacy allele was associated with an estimated 1.6-month decrease in age at pubertal onset (*P*-trend = 0.06), and girls in the group with 5–6 efficacy alleles presented with a lower age at puberty compared with the group with 0–2 efficacy alleles (*P* = 0.02), indicating a possible additive effect on pubertal timing. In a sub-analysis restricted to rs727479 and rs2899472, a higher combined efficacy allele count was significantly associated with earlier pubertal onset. Each additional efficacy allele was associated with an approximately 24% shorter time to B2 (*P* < 0.01).

No difference was found in the E2/T ratio with increasing number of efficacy alleles (*P*-trend = 0.16).

In a sensitivity analysis including circulating LH concentrations in the interval-censored model of pubertal onset, LH was strongly associated with earlier pubertal onset (*P* < 0.01). Adjustment for LH did not abolish the association between *CYP19A1* efficacy allele groups and pubertal timing, and girls in the highest efficacy group (5–6 alleles) continued to demonstrate significantly earlier pubertal onset (*P* = 0.02).

Using interval-censored survival regression, we found no significant associations between *CYP19A1* genotypes (rs727479, rs2899472 and rs10046) and age at menarche in girls (all *P* > 0.09, data not shown).

#### Boys

Overall, we observed no statistically significant associations between *CYP19A1* genotypes and pubertal timing in boys (Figs 2 and 3).

### Effects of reproductive hormones and *CYP19A1* genotypes on pubertal gynecomastia

**Figure 4:**
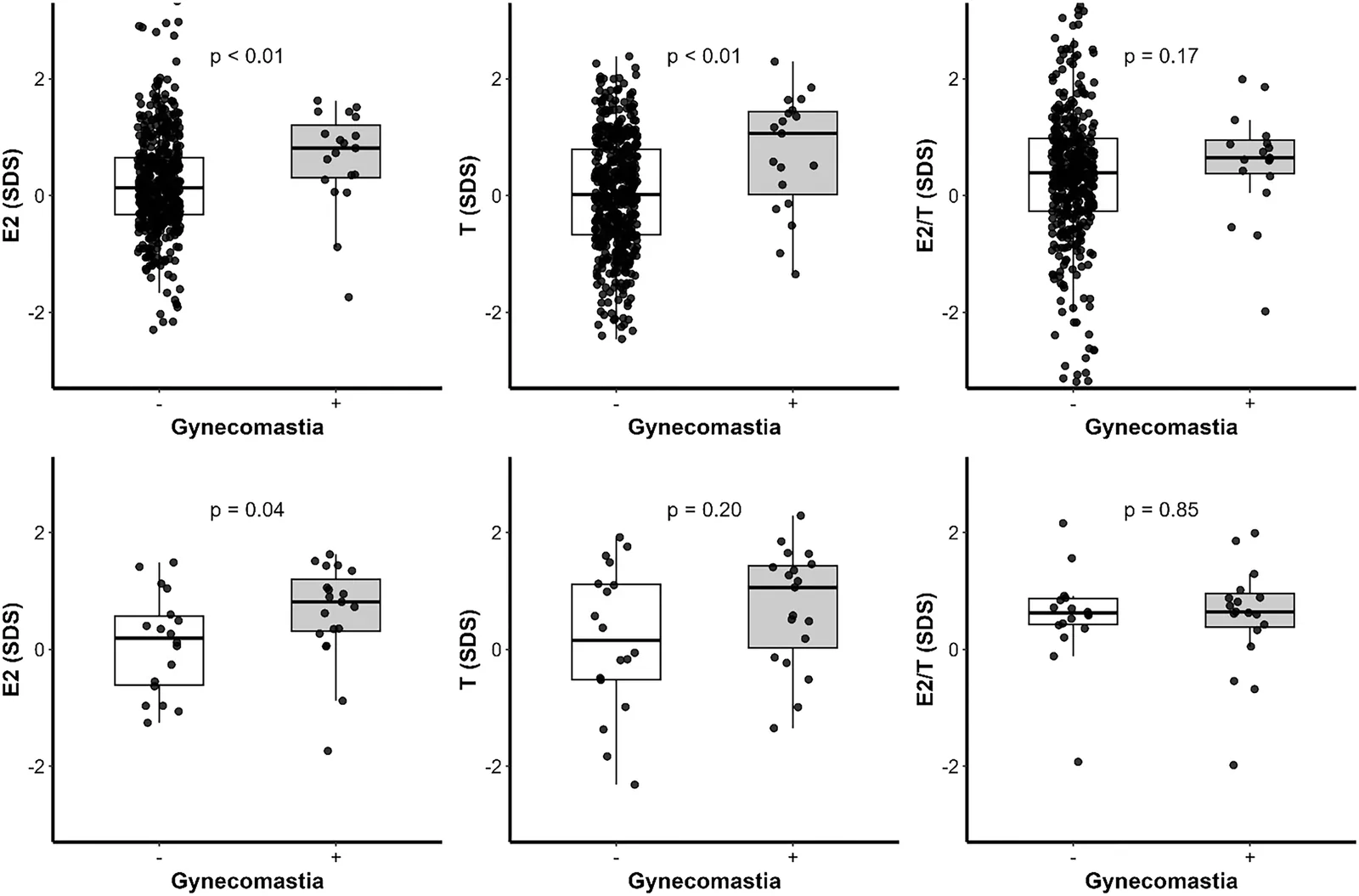
Concentrations of E2 and T and the E2/T ratio in healthy boys with and without gynecomastia. Serum concentrations of estradiol (E2, SDS) and testosterone (T, SDS) and the E2/T ratio (SDS) are shown for boys with (+, *n* = 19) and without (−, *n* = 480) gynecomastia. The upper panel shows the full cohort; group differences were assessed using the Wilcoxon rank-sum test. The lower panel shows results from an age-matched 1:1 analysis (*n* = 19 matched pairs); group differences in the matched sample were assessed using weighted linear models. Each point represents an individual observation. The boxes indicate median and interquartile range.

A total of 19 boys (4.5%) presented with pubertal gynecomastia at the time of examination. All 19 boys (mean age: 13.4 years) had testis volumes > 3 mL. As shown in Fig. 4, boys with gynecomastia had significantly higher serum concentrations of both E2 and T (SDS) compared with those without (both *P*-values <0.01), whereas the E2/T ratio did not differ between the groups (*P* = 0.17).

In the age-matched sensitivity analysis comparing boys with gynecomastia to those without (the lower bar), E2 (SDS) remained significantly higher in boys with gynecomastia, whereas the difference in T (SDS) was no longer significant (Fig. 4).

**Figure 5:**
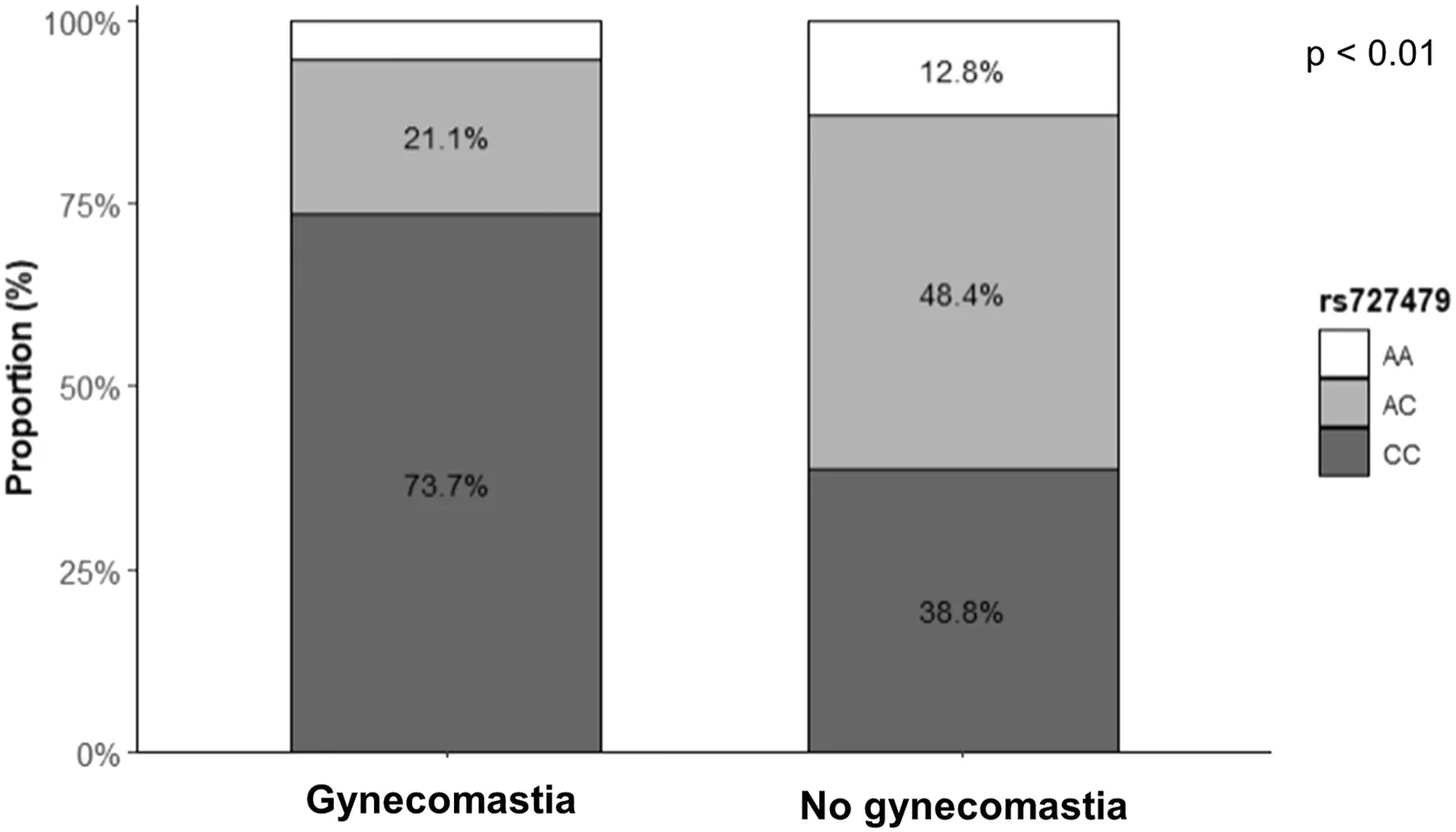
Genotype distribution of rs727479 in boys with and without gynecomastia. A stacked bar chart showing the relative distribution of AA, AC and CC genotypes in boys with gynecomastia (*n* = 19) and without gynecomastia (*n* = 480). Group differences were assessed using Fisher’s exact test (*P* < 0.01).

The rs727479 CC genotype was more frequent in boys with gynecomastia compared with those without (73.7 vs 38.8%, respectively, *P* < 0.01) (Fig. 5). For rs2899472 and rs10046, no difference in the presence of gynecomastia between genotypes was seen.

## Discussion

In this population-based study of healthy school children and adolescents, we evaluated genetic variants in the *CYP19A1* gene and their possible effects on pubertal timing in boys and girls and on development of gynecomastia in boys. We found no association between *CYP19A1* genotypes and circulating concentrations of E2 and T or the E2/T ratio. However, specific *CYP19A1* variants (rs727479 and rs2899472) were associated with earlier pubertal onset in girls. In boys, the *CYP19A1* genotype was not associated with pubertal onset, whereas *CYP19A1* variants were associated with gynecomastia, accompanied by higher serum E2 concentrations.

### Impact of *CYP19A1* variants on circulating concentrations of E2 and T and the E2/T ratio

The absence of an association between *CYP19A1* genotypes and circulating sex steroid concentrations does not exclude a functional role of these variants in pubertal development. Aromatase is expressed in multiple extra-gonadal tissues, including breast tissue, adipose tissue, bone and brain, where it is regulated locally through tissue-specific promoters that may not be reflected in circulating estrogen concentrations (44). Thus, variants such as rs727479 and rs2899472 may influence local estrogen biosynthesis in target tissues, including breast tissue, without resulting in detectable changes in circulating E2 levels. This interpretation is supported by the high proportion of E2 values at or below the limit of detection in our cohort (36% in boys; 11% in girls), which may have reduced our ability to detect genotype–hormone associations. Similarly, rs10046, located in the 3′ UTR of *CYP19A1*, may influence post-transcriptional regulation of aromatase expression, representing a complementary tissue-level mechanism.

### *CYP19A1* SNPs and pubertal onset

Girls carrying the AA genotype in both rs727479 and rs2899472 demonstrated weak trends toward earlier pubertal onset (4.4 and 6.4 months earlier than CC genotypes, respectively). In a combined efficacy allele model including all three SNPs, pubertal onset decreased by ∼1.6 months per additional allele. In a sub-analysis restricted to rs727479 and rs2899472, combined efficacy allele score was associated with earlier pubertal onset, highlighting that these two variants provide the most robust evidence for an effect on pubertal timing in girls, while the inclusion of rs10046 appears to attenuate the overall association. This may reflect the more ambiguous functional role of rs10046, where this variant is thought to influence aromatase expression post-transcriptionally, yet its direction of effect on circulating E2 has been inconsistent across menopausal populations (30, 45).

Prior research on *CYP19A1* has applied additive allele models in pharmacogenetic studies of aromatase inhibitor response in breast cancer, where carriers of efficacy alleles show different treatment outcomes (25). At the population level, large GWASs of pubertal timing have documented additive allele effects across thousands of independent loci (3). It should be noted that the allele classifications were originally derived from data in postmenopausal women and the assumption of equivalent directionality in children has not been independently validated. The current findings in a pediatric context should therefore be considered exploratory and hypothesis-generating.

Together, the absence of an association between these SNPs and age at menarche and the persistence of the association with pubertal onset after adjustment for LH suggest that *CYP19A1* variation may influence early estrogen-sensitive pubertal changes. Even though evidence in pediatric populations is still limited, our findings support previous studies reporting an association between the A allele of rs727479 and elevated circulating concentrations of E2 (24, 25). Similarly, Lee *et al.* (23) identified a link between TTTA repeat length polymorphisms in *CYP19A1* and central precocious puberty in girls, supporting a role for aromatase in pubertal regulation.

We found no effect of *CYP19A1* genotypes on pubertal onset in healthy boys, suggesting that common genetic variations in *CYP19A1* are not major determinants of normal pubertal timing. In contrast, aromatase deficiency can affect hormone regulation and pubertal progression as illustrated by the case reported by Costanzo *et al.*, in which a boy exhibited accelerated pubertal progression and elevated serum testosterone (46). In accordance with the absence of hormone-level associations, height SDSs did not differ across *CYP19A1* genotypes in our cohort. This is in keeping with the observation that only the complete or near-complete aromatase deficiency leads to the well-described phenotype of impaired epiphyseal fusion and tall stature in males. Common SNPs with modest effects on aromatase activity are thus unlikely to affect linear growth.

### *CYP19A1* SNPs and hormone levels in boys with gynecomastia

In our cohort, 19 (4.5%) boys presented with pubertal gynecomastia. Contrary to previous studies, including one by Reinehr *et al.*, which reported higher E2/T levels in boys with gynecomastia (primarily due to reduced T levels), we observed significantly higher E2 and T in affected boys, without an elevated E2/T ratio (33, 34). This suggests a simultaneous upregulation of estrogenic and androgenic activity, possibly reflecting transient HPG axis activation rather than relative estrogen excess. Because SDSs may be disproportionately skewed in the youngest boys, where reference intervals are narrow, we performed a supplementary age-matched 1:1 analysis. This confirmed higher E2 concentrations in boys with gynecomastia, whereas the difference in T was no longer statistically significant, and the E2/T ratio remained unchanged. These results are consistent with previous studies, supporting the notion that estrogen excess, rather than reduced androgen concentrations, is the primary driver of pubertal gynecomastia.

Our finding of an overrepresentation of the rs727479 CC genotype in boys with gynecomastia may indicate a role for aromatase-related genetic variation in modulating estrogen production during puberty. No significant associations were found for rs2899472 or rs10046. The low prevalence of gynecomastia (4.5%) likely reflects the transient nature of this phenomenon rather than its true frequency. The cross-sectional nature of our study will evidently include boys without gynecomastia at examination who already have had gynecomastia, or who may develop gynecomastia at a later time point. Thus, our control population of boys without gynecomastia may be inflated by cases who will develop gynecomastia at another time point, hereby reinforcing a potential genetic association in the boys with gynecomastia.

### Allele distribution

We observed an allele distribution of rs727479 that differed from reference populations in both sexes, where the C allele has been reported as the minor allele. The supplier of the *CYP19A1* primers (LGC Genomics) confirmed the validity of the reagents used, and no irregularities concerning the DNA testing plates were detected. Thus, although somewhat unexpected, we consider this finding robust, and it may reflect a true population-specific difference in allele distribution.

### Strengths and limitations

This study benefits from several strengths. It is based on the second Copenhagen Puberty Study including otherwise healthy school children from the background population. Standardized clinical assessments and high-quality hormonal measurements improve data reliability, and the selected *CYP19A1* variants are supported by prior functional evidence, strengthening biological plausibility.

However, the cross-sectional design precludes causal inference, and subgroup analyses, particularly in boys with gynecomastia, are limited by low numbers. We only included boys with the presence of gynecomastia at the time of examination, and all the rest served as controls without gynecomastia. However, the latter group may already have had gynecomastia in the past or develop it in the future. Thus, statistical power was limited, and larger sample sizes might have altered significance levels of observed effects. Finally, although pubertal staging was based on validated methods, interval censoring introduces uncertainty in pinpointing the exact timing of onset.

We evaluated breast development as a marker of pubertal onset in girls; however, breast development alone does not allow for differentiating between local estrogen activation in breast tissue from central HPG activation. Thelarche can occur without gonadotropin stimulation, as seen in transient thelarche (47). The persistence of the *CYP19A1* efficacy allele association after LH adjustment supports this gonadotropin-independent mechanism. Consistent with this interpretation, no association was found between *CYP19A1* genotypes and age at menarche, which, unlike thelarche, requires sustained central HPG axis activation. This is consistent with previous studies demonstrating that genetic variants may influence the timing of thelarche without affecting age at menarche (48).

## Conclusion

In this cohort of healthy Danish children, we observed that genetic variation in *CYP19A1* may be associated with the timing of pubertal onset in girls. Girls carrying the AA genotypes of rs727479 and rs2899472 exhibited an effect on earlier age at pubertal onset, and a combined efficacy allele model indicated a progressively lower pubertal age with increasing number of efficacy alleles. In boys, an association was observed between the rs727479 CC genotype and the presence of gynecomastia, pointing to a potential role for aromatase activity in pubertal breast tissue development of boys.

Furthermore, boys with gynecomastia presented with higher E2 and T concentrations measured by LC–MS/MS than those without, but without a corresponding increase in the E2/T ratio. In the age-matched analysis, only E2 remained elevated, supporting the role of increased estrogen as a primary driver in pubertal gynecomastia.

## Declaration of interest

The authors declare that there is no conflict of interest that could be perceived as prejudicing the impartiality of the work reported.

## Funding

This study was supported by the Kirsten and Freddy Johansen Foundation and the Danish Medical Research Council (grant no. 271-05-0337).

## Data availability

Some or all datasets generated during and/or analyzed during the current study are not publicly available but are available from the corresponding author on reasonable request.

## Ethical approval

This study was approved by the Committees on Health Research in the Capital Region of Denmark. Journal number: H-KF-282214.
